# Interoceptive abnormalities in COPD patients: Their predictive role in anxiety and acute exacerbation of COPD

**DOI:** 10.1097/MD.0000000000043023

**Published:** 2025-07-04

**Authors:** Ming-wan Su, Li-kun Ge, Xiao-yan Yao, Hao-xiang Zhang, Ying-zi Tian, Yu-jin Ding, Yan-yi Liu, Xiao Xia, Xiao-hong Liu, Ting Fang, Gao-xia Wei, Guang-xi Li

**Affiliations:** a Guanganmen Hospital, China Academy of Chinese Medical Sciences, Beijing, China; b State Key Laboratory of Cognitive Science and Mental Health, Institute of Psychology, Chinese Academy of Sciences, Beijing, China; c Department of Psychology, University of Chinese Academy of Sciences, Beijing, China.

**Keywords:** a cross-sectional study, anxiety sensitivity, chronic obstructive pulmonary disease, healthcare utilization, interoceptive abnormalities, psychological comorbidities

## Abstract

Chronic obstructive pulmonary disease (COPD) is a leading cause of morbidity and mortality worldwide, significantly impacting both physical and psychological health. Anxiety, a prevalent comorbidity in COPD, exacerbates respiratory symptoms, impairs disease management, and increases healthcare utilization. Interoceptive sensitivity, the ability to perceive and interpret internal bodily signals, plays a critical role in managing chronic conditions, yet its influence on healthcare utilization in COPD remains underexplored. This study investigates the relationship between interoceptive sensitivity, anxiety sensitivity, and acute exacerbation hospitalization rates in COPD patients, aiming to identify potential therapeutic targets to improve patient outcomes. A cross-sectional study was conducted among 73 COPD patients recruitedboth outpatients and inpatients of respiratory department. Interoceptive sensitivity was assessed using a body perception questionnaire-very short form scale, and autonomic nervous system (ANS) reactivity was assessed through body perception questionnaire 20-autonomic nervous system. Anxiety sensitivity was measured with the anxiety sensitivity index-3 physical concerns subscale. Depression and anxiety were evaluated by PHQ-9 and GAD-7 seperately. Acute exacerbation of COPD (AECOPD) was identified by self-reported hospitalizations and verified via medical records over the preceding year, according to GOLD 2023 criteria. Logistic regression models were used to analyze the associations between interoceptive sensitivity, anxiety sensitivity, and its correlation with AECOPD. Higher interoceptive sensitivity was associated with a 28.4% reduction in the likelihood of AECOPD (odds ratios = 0.716, 95% confidence intervals: 0.58–0.88, *P* = .002), suggesting a protective effect. Conversely, elevated anxiety sensitivity increased AECOPD by 126.6% (odds ratios = 2.266, 95% confidence intervals: 1.32–3.89, *P* = .003). Other demographic and clinical factors, including age, body mass index, and COPD duration, were not significant predictors of AECOPD. The findings reveal the dual role of interoceptive sensitivity and anxiety sensitivity in influencing healthcare utilization among COPD patients. Interventions targeting interoceptive skills and anxiety sensitivity, such as mindfulness-based therapy, may improve symptom management, reduce emergency healthcare utilization and hospital admission, and enhance overall quality of life in this population. Future research should explore longitudinal and interventional studies to further elucidate these relationships.

## 1. Introduction

Chronic obstructive pulmonary disease (COPD) is a leading cause of morbidity and mortality worldwide, characterized by persistent respiratory symptoms, airflow limitation, and systemic inflammation. While COPD primarily manifests as a physical condition, it is often accompanied by significant psychological comorbidities, including anxiety and depression, which exacerbate disease burden and impair quality of life.^[1,2]^ Anxiety is prevalent in approximately 50% of COPD patients and is significantly associated with increased frequency of acute exacerbation, healthcare utilization, hospitalization, reduced treatment adherence, and worsened clinical outcomes.^[3]^

Interoception, defined as the ability to perceive and interpret internal bodily states, plays a critical role in managing chronic diseases, including COPD. Interoceptive dysfunction, whether as reduced awareness of bodily sensations or heightened sensitivity to them, can impair symptom management and amplify psychological distress. For instance, patients with diminished interoceptive sensitivity may fail to recognize early exacerbation symptoms, delaying treatment, while those with excessive interoceptive sensitivity may misinterpret benign sensations, such as mild dyspnea, as life-threatening.^[4,5]^

The neural mechanisms underlying interoception and its role in COPD have been increasingly studied. Herigstad^[3]^ demonstrated that dyspnea-related cues engage specific regions of the prefrontal cortex, suggesting a neurophysiological basis for symptom perception and regulation in COPD. Furthermore, heightened interoceptive sensitivity has been linked to increased anxiety sensitivity, defined as the fear of anxiety-related bodily sensations, forming a detrimental cycle of symptom misinterpretation, psychological distress, and excessive healthcare resource utilization.^[6,7]^

Mindfulness-based interventions, which enhance interoceptive sensitivity, have emerged as promising therapeutic approaches for managing the psychological dimensions of chronic diseases, including COPD. These interventions are associated with improved symptom interpretation, reduced anxiety sensitivity, and better overall disease management.^[1]^ However, despite these advances, the relationship between interoceptive sensitivity, anxiety sensitivity, and healthcare utilization in COPD remains underexplored.

This study aims to investigate the role of interoceptive sensitivity and anxiety sensitivity in predicting hospitalization rates among COPD patients. By examining the interplay between these factors, this research seeks to identify potential therapeutic targets that address both the physical and psychological dimensions of COPD, ultimately improving patient outcomes and reducing healthcare burdens.

## 2. Materials and methods

This study employed a cross-sectional design to examine the relationships between interoceptive sensitivity, anxiety sensitivity, and healthcare utilization in patients diagnosed with COPD. The study was conducted at Guang’anmen Hospital in Beijing, China, between January and December 2023. This study adhered to the Declaration of Helsinki principles and received ethical approval from the Ethics Committee of the Guang’anmen Hospital, China Academy of Chinese Medical Sciences, (2022-111-KY). All participants were informed of the study’s objectives. Data confidentiality and anonymity were maintained throughout the study.

## 3. Study population

Patients were recruited voluntarily from the respiratory department of Guang’anmen Hospital. Inclusion criteria were as follows: confirmed clinical diagnosis of COPD based on GOLD (Global Initiative for Chronic Obstructive Lung Disease) guidelines; age 40 years or older; ability to complete self-report questionnaires independently or with assistance. Exclusion criteria included: severe comorbidities likely to affect interoceptive processing (e.g., advanced cardiac or neurological disorders); cognitive impairments interfering with the accuracy of self-reported data; recent acute exacerbations within the preceding 4 weeks.

## 4. Measurements and data collection

### 4.1. Interoceptive sensitivity

Interoceptive sensitivity was assessed using 2 subscales of the Body Perception Questionnaire (BPQ). The body perception questionnaire body awareness very short form evaluates self-reported sensitivity to bodily sensations (e.g., breathlessness). The body perception questionnaire autonomic symptoms short form (body perception questionnaire 20-autonomic nervous system) measures ANS reactivity, serving as a physiological indicator of sensitivity.. The items of the ANS reactivity subscale clustered into 2 factors, named supradiaphragmatic reactivity and subdiaphragmatic reactivity.^[8]^ Patients rated their ability to perceive and interpret physiological states, such as breathlessness, heart rate, and bodily sensations. The scores provided an overall measure of interoceptive sensitivity, with higher scores indicating greater sensitivity.

### 4.2. Anxiety sensitivity

Anxiety sensitivity was measured with the anxiety sensitivity index-3 physical concerns subscale. This validated self-report tool evaluates the degree to which individuals fear anxiety-related bodily sensations (e.g., increased heart rate or shortness of breath). This scale specifically assesses on somatic concerns highly relevant to COPD patients.

### 4.3. Healthcare utilization

Healthcare utilization data were collected through retrospective medical record reviews and prospective self-reports to identify emergency healthcare utilization and hospital admission events due to acute exacerbations of COPD (AECOPD) over the preceding 12 months. AECOPD was operationalized as a dichotomous variable (0 = no events; 1 = at least 1 hospitalization or emergency department visit). This composite metric served as a proxy for acute exacerbation severity and healthcare resource utilization.

### 4.4. Data collection procedure

Data collection involved a combination of clinical interviews, self-administered questionnaires, and medical record reviews. Trained research assistants facilitated the process to ensure accuracy and completeness. Baseline demographic and clinical characteristics, including age, gender, body mass index (BMI), smoking history, and duration of COPD, were also recorded.

## 5. Statistical analysis

All statistical analyses were performed using SPSS (Version 26.0, https://www.ibm.com/). Descriptive statistics were used to summarize demographic and clinical characteristics. Continuous variables were expressed as means ± standard deviations (SD), while categorical variables were presented as frequencies and percentages.

Binary logistic regression models were utilized to examine the associations between interoceptive sensitivity, anxiety sensitivity, and AECOPD. The dependent variable, AECOPD, was defined dichotomously. Independent variables included interoceptive sensitivity and anxiety sensitivity (measured by anxiety sensitivity index-3 physical concerns subscale), with adjustments for potential confounders, including age, gender, BMI, smoking history, and COPD duration. Odds ratios (OR) and 95% confidence intervals (CI) were computed to quantify effect sizes, with statistical significance set determined at *P* < .05.

## 6. Results

### 6.1. Baseline characteristics

A total of 73 COPD patients were included in the study. The mean age of the participants was 65.85 ± 8.76 years, with the majority being male (58 males, 15 females). The average BMI was 23.49 ± 3.77 kg/m², and the mean disease duration was 8 years. Approximately 68.5% of patients reported hospitalization in the past 1 year, in which 45.2% reported once hospitalization, 16.4% reported twice hospitalizations, and 6.8% reported 3 times or more. Regarding emergency visits, 80.8% had none, while 15.1% and 4.1% reported once and twice or more visits, respectively. Table 1 provides detailed demographic and clinical characteristics.

**Table 1:** Demographic and clinical characteristics of the study population.

Characteristic	Patients (n = 73)
Mean age (yr)	65.85 ± 8.76
Gender	58 males, 15 females
BMI	23.49 ± 3.77
Mean disease duration (yr)	8
Hospitalizations in the past 1 yr	
0 (n, %)	23 (31.5)
1 (n, %)	33 (45.2)
2 (n, %)	12 (16.4)
≥3 (n, %)	5 (6.8)
Emergency visits in the past 1 yr	
0 (n, %)	59 (80.8)
1 (n, %)	11 (15.1)
≥2 (n, %)	3 (4.1)

BMI = body mass index.

### 6.2. Relationships between interoceptive sensitivity, anxiety sensitivity, and AECOPD

Multivariate logistic regression revealed significant relationships between interoceptive awareness, anxiety sensitivity, and AECOPD. The primary results are summarized in Table 2.

**Table 2:** The binary logistic regression analysis of acute exacerbations.

	OR	*X* ^2^	*P* value	CI
Age	1.063	2.00	.157	0.98–1.16
BMI	1.097	1.07	.301	0.92–1.31
Gender	0.377	0.98	.323	0.05–2.60
Smoking history	0.999	0.00	.979	0.96–1.04
COPD disease course	1.001	0.02	.890	0.99–1.01
BPQ awareness	0.716	9.92	.002	0.58–0.88
BPQ ANS	0.885	3.09	.079	0.77–1.01
ASI-P	2.266	8.78	.003	1.32–3.89

ANS = autonomic nervous system, BMI = body mass index, BPQ = body perception questionnaire, COPD = chronic obstructive pulmonary disease.

#### 6.2.1. Interoceptive sensitivity

**Figure 1.**
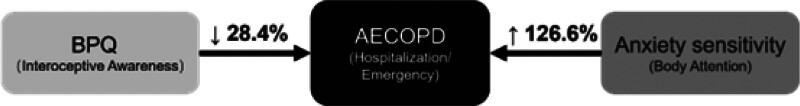
Conceptual model: relationship between interoception, anxiety, and healthcare needs in COPD. This figure illustrates the feedback loop in which interoceptive dysfunction and anxiety sensitivity contribute to increased healthcare needs in COPD. COPD = chronic obstructive pulmonary disease.

Higher interoceptive sensitivity significantly reduced the likelihood of experiencing AECOPD. Specifically, each unit increase in interoceptive sensitivity corresponded to a 28.4% reduction in the odds of AECOPD (OR = 0.716, 95% CI: 0.58–0.88, *P* = .002; Fig. 1). This finding suggests that improved sensitivity awareness of bodily signals may enable patients to better manage symptoms and reduce unnecessary healthcare utilization.

#### 6.2.2. Anxiety sensitivity

Conversely, anxiety sensitivity was strongly in association with healthcare utilization. A 1-unit increase in anxiety sensitivity was associated with a 126.6% increase in the odds of AECOPD (OR = 2.266, 95% CI: 1.32–3.89, *P* = .003; calculated as [2.266–1] × 100%). These results highlight the detrimental impact of heightened anxiety sensitivity on COPD management, potentially leading to increased healthcare utilization due to misinterpretation of symptoms.

#### 6.2.3. Covariates

Age, BMI, gender, and smoking history were not significantly associated with healthcare utilization in the multivariate model. COPD duration similarly showed no significant relationship with AECOPD.

## 7. Discussion

The current study highlights a significant relationship between interoceptive sensitivity and healthcare utilization in patients with chronic obstructive pulmonary disease. Patients exhibiting higher interoceptive sensitivity experienced a 28.4% reduction in healthcare utilization. This finding underscores the potential protective role of interoceptive sensitivity in managing chronic conditions.

Interoceptive sensitivity refers to the ability to perceive and interpret internal bodily states, such as changes in breathlessness or heart rate. In COPD patients, interoceptive dysfunction may lead to a misinterpretation of these signals, escalating mild symptoms into perceived emergencies. Conversely, patients with heightened interoceptive sensitivity can better distinguish between benign and critical symptoms, thus avoiding unnecessary healthcare utilization. These findings are consistent with Mehling et al,^[9]^ who identified the positive role of interoceptive sensitivity in promoting self-regulation and symptom management in chronic illnesses.

Often, COPD is accompanied by heightened anxiety sensitivity, defined as the fear of anxiety-related bodily sensations. This study found that patients with higher anxiety sensitivity demonstrated a 96.3% increase in healthcare utilization. The interplay between anxiety sensitivity and interoceptive dysfunction provides a compelling explanation for this phenomenon. Anxiety-prone individuals are more likely to perceive normal physiological variations, such as transient breathlessness, as severe health threats. This hypervigilance exacerbates both psychological distress and healthcare utilization, creating a self-perpetuating cycle.

Davenport et al^[10]^ provided evidence of altered sensory processing in COPD patients, where central nervous system pathways amplify interoceptive signals, leading to heightened sensitivity to respiratory sensations. This central amplification may explain why COPD patients with anxiety are more likely to overreact to minor changes in their symptoms. Additionally, Meuret et al^[11]^ demonstrated that anxiety sensitivity significantly predicts the misinterpretation of bodily sensations, further supporting the hypothesis that interoceptive dysfunction exacerbates healthcare-seeking behaviors.

Interoceptive dysfunction in COPD may be rooted in the disease’s pathophysiological characteristics. The chronic inflammation and systemic hypoxia associated with COPD can impair neural pathways responsible for interoceptive processing. Moreover, respiratory mechanical constraints, such as airflow obstruction and hyperinflation, alter the body’s internal signaling mechanisms, contributing to distorted interoceptive feedback. Davenport et al^[10]^ noted that chronic dyspnea may rewire central sensory pathways, leading to persistent hypersensitivity to respiratory signals.

Psychological comorbidities, particularly anxiety and depression, are prevalent in COPD and closely linked to interoceptive dysfunction. Anxiety amplifies sensitivity to bodily signals, while depression can dull interoceptive sensitivity, both of which impair effective symptom recognition and management.^[12]^ These findings suggest a bidirectional relationship between psychological states and interoceptive processing, where each factor exacerbates the other.

Age, socioeconomic status, and health literacy also play critical roles. Older COPD patients with multiple comorbidities may struggle to differentiate between symptoms of COPD and those of other conditions, such as heart failure or anxiety disorders. Additionally, previous research emphasized that low health literacy limits patients’ ability to understand their symptoms, increasing reliance on emergency services.^[13]^

The findings of this study align with previous research on interoception in respiratory diseases. For instance, in asthma patients, it was observed that heightened interoceptive sensitivity led to an overestimation of breathlessness severity, even when objective measures of airway obstruction were mild.^[10]^ Similar patterns have been reported in irritable bowel syndrome (IBS), where interoceptive dysfunction correlates with increased healthcare utilization and psychological distress.^[14,15]^

Conversely, interoceptive training has shown significant promise in improving patient outcomes.^[16]^ Mindfulness-based stress reduction programs was suggesting to enhance interoception and reduced anxiety sensitivity in patients with chronic pain. Similar report addressed that mindfulness-oriented interventions helped COPD patients better manage dyspnea, reducing the frequency of emergency visits.^[17]^ These findings suggest that targeted interoceptive interventions could offer cross-condition benefits, particularly in diseases with high psychological and physiological interplay.

Previous research had emphasized the importance of interoceptive sensitivity in chronic disease management. Patients with improved interoceptive skills are better equipped to interpret and respond to their symptoms, reducing healthcare utilization.^[9,18]^ Also, anxiety sensitivity emerges as a key factor driving overutilization of healthcare resources across chronic conditions.^[19,20]^ Accordingly, it was noted that cognitive-behavioral interventions targeting anxiety sensitivity were effective in breaking the cycle of symptom misinterpretation and excessive medical care.^[11,21,22]^ Interventions like mindfulness-based stress reduction and cognitive-behavioral therapy have shown consistent success in enhancing interoceptive sensitivity and reducing anxiety-related behaviors.^[2,21,22]^ Long-term benefits of these therapies include reduced hospitalization rates and improved quality of life.^[14,23–25]^

Despite its contributions, the study has several limitations. First, this cross-sectional study precludes causal inferences. Longitudinal research should be preferred to clarify the directionality of relationships between interoception, anxiety sensitivity, and hospitalization. Secondly, self-reported data could introduces potential bias to our result. Objective assessments, such as respiratory biofeedback or heart rate variability monitoring, would provide more reliable insights into interoceptive sensitivity. Nevertheless, the sample size of our study could limit the generalizability of the findings. Future studies should include larger and more diverse populations to validate the results across different demographic groups and healthcare settings. While the study focuses on anxiety sensitivity, it did not consider other psychological factors, such as depression or health-related quality of life, which may influence the observed relationships.

## 8. Conclusion

This study reveals the significant role of interoceptive sensitivity and anxiety sensitivity in influencing acute exacerbation among COPD patients. Targeted interventions aimed at enhancing interoceptive skills and reducing anxiety sensitivity, such as mindfulness-based therapies and cognitive-behavioral therapy, hold promise for improving COPD management. Future research should focus on integrating interoceptive training into routine COPD care, using longitudinal designs and objective measures to validate these findings. Additionally, multidisciplinary care models that address both the physical and psychological dimensions of COPD could significantly improve patient outcomes and reduce the burden on healthcare systems.

## Acknowledgments

We thank the patients for cooperation who participated in this study.

## Author contributions

**Conceptualization:** Guang-xi Li.

**Formal analysis:** Guang-xi Li, Ming-wan Su, Li-kun Ge, Hao-xiang Zhang.

**Funding acquisition:** Guang-xi Li, Gao-xia Wei.

**Investigation:** Ming-wan Su, Li-kun Ge, Xiao-yan Yao, Hao-xiang Zhang, Ying-zi Tian, Yu-jin Ding, Yan-yi Liu, Xiao Xia, Xiao-hong Liu, Ting Fang.

**Project administration:** Guang-xi Li.

**Supervision:** Guang-xi Li, Gao-xia Wei.

**Visualization:** Guang-xi Li.

**Writing – original draft:** Guang-xi Li, Ming-wan Su, Li-kun Ge, Xiao-yan Yao, Hao-xiang Zhang, Ying-zi Tian, Yu-jin Ding, Yan-yi Liu, Xiao Xia, Xiao-hong Liu.

**Writing – review & editing:** Guang-xi Li, Ming-wan Su, Li-kun Ge, Xiao-yan Yao, Hao-xiang Zhang, Ying-zi Tian, Yu-jin Ding, Yan-yi Liu, Xiao Xia, Xiao-hong Liu, Ting Fang, Gao-xia Wei.
